# Clinicopathological analysis of pulmonary mucoepidermoid carcinoma

**DOI:** 10.1186/1477-7819-12-33

**Published:** 2014-02-08

**Authors:** Cheng Shen, Guowei Che

**Affiliations:** 1 Department of Cardiovascular and Thoracic Surgery, West China Hospital, Sichuan University, No. 37, Guoxue Alley, Chengdu, Sichuan Province 610041, China

**Keywords:** Mucoepidermoid carcinoma, Lung, Surgery, Pathological histology

## Abstract

**Background:**

Mucoepidermoid carcinoma (MEC) of the lung is a rare malignant neoplasm. We aimed to investigate clinicopathological features, therapies, and prognoses of eight MEC cases.

**Methods:**

Eight patients underwent surgical treatment for pulmonary MEC between 2005 and 2012 at the Thoracic Surgical Department of West China Hospital, Sichuan, China. The clinical data, radiological manifestation, treatment strategy, pathological findings, and prognoses of all patients were analyzed retrospectively.

**Results:**

Among the eight cases (four males and four females), the age of patients ranged from 35 to 71 years (mean age 50.67 years). Two tumors were located in the upper lobes and three masses were located in the lower lobes. The other three lumps were located in the left main bronchus, middle segmental bronchus of the right lobe, and trachea, respectively. The characteristics of the tumors were consistent with low grade MEC (n = 6) and high grade MEC (n = 2). All of the patients were sent for oncological evaluations, and three patients with N1 or N2 disease received chemotherapy. One of the patients died from brain metastasis at 15 months. Seven of the eight patients were alive at the time of evaluation. The median survival time was 40 (range 8 to 88) months.

**Conclusion:**

Mucoepidermoid tumors have to be treated by radical surgery with lymph node sampling and dissection. Patients with low grade tumors can be expected to be cured following complete resection. Careful histological typing plays a key role in prediction of late results, and further studies are needed.

## Background

Mucoepidermoid carcinoma (MEC) of the lung is a tumor of low malignant potential of bronchial gland origin, which is characterized by the presence of squamoid cells, mucin-secreting cells, and cells of intermediate type [1]. MEC and adenoid cystic carcinoma are both considered to be salivary gland-type neoplasms. It usually arises in the parotid and submandibular salivary glands and in the minor salivary glands of the oral cavity and perimaxillary region. However, MEC is not common in the lungs, particularly in children, accounting for only 0.1% to 0.2% of primary lung cancers [2-5]. This study evaluated the results of eight cases that underwent surgery for MEC.

## Methods

### Patients and clinical data

Between 2005 and 2012, eight patients were operated on for pulmonary mucoepidermoid tumors at the Thoracic Surgical Department of West China Hospital, Sichuan, China. These tumors accounted for 0.11% of all resected pulmonary neoplasms in that period. The patients were evaluated in terms of their age, gender, symptoms, diagnostic approaches, surgical methods, and follow-up findings. Symptoms such as hemoptysis, cough, dyspnea, and chest pain were dominated in seven patients and only one patient was free of symptoms, detected accidentally.

All of the patients underwent routine laboratory studies, respiratory function tests, electrocardiography, chest X-ray, computed tomography (CT) of the thorax, brain CT or magnetic resonance imaging (MRI), and abdominal CT. Fiberbronchoscopy was undertaken in six cases. In the preoperative period, three patients had a diagnosis of MEC, whereas the other five patients were diagnosed with non-small-cell lung cancer (NSCLC). Complete mass resection with lobectomy or bronchial sleeve resection was undertaken in all patients by a standard posterolateral thoracotomy incision or by video-assisted thoracoscopic surgery (VATS). All of the tumors were staged postoperatively according to the seventh international TNM staging system. The diagnosis was verified immunohistochemically.

All patients underwent clinical and radiological follow-up for a median of 40 (range 8 to 88) months. All patients were assessed quarterly for the first 2 years with a history, physical examination, and chest X-ray. Laboratory tests and advanced radiological methods were requested if there were any symptoms. Additionally, all patients or the families were asked by telephone when the study was performed and asked about any signs of recurrence or complications.

### Statistical analysis

The statistical analysis was performed using IBM SPSS Statistics, version 16.0 (IBM Corporation, Armonk, NY, USA). Descriptive analyses and Kaplan - Meier survival analysis were used.

## Results

**Figure 1:**
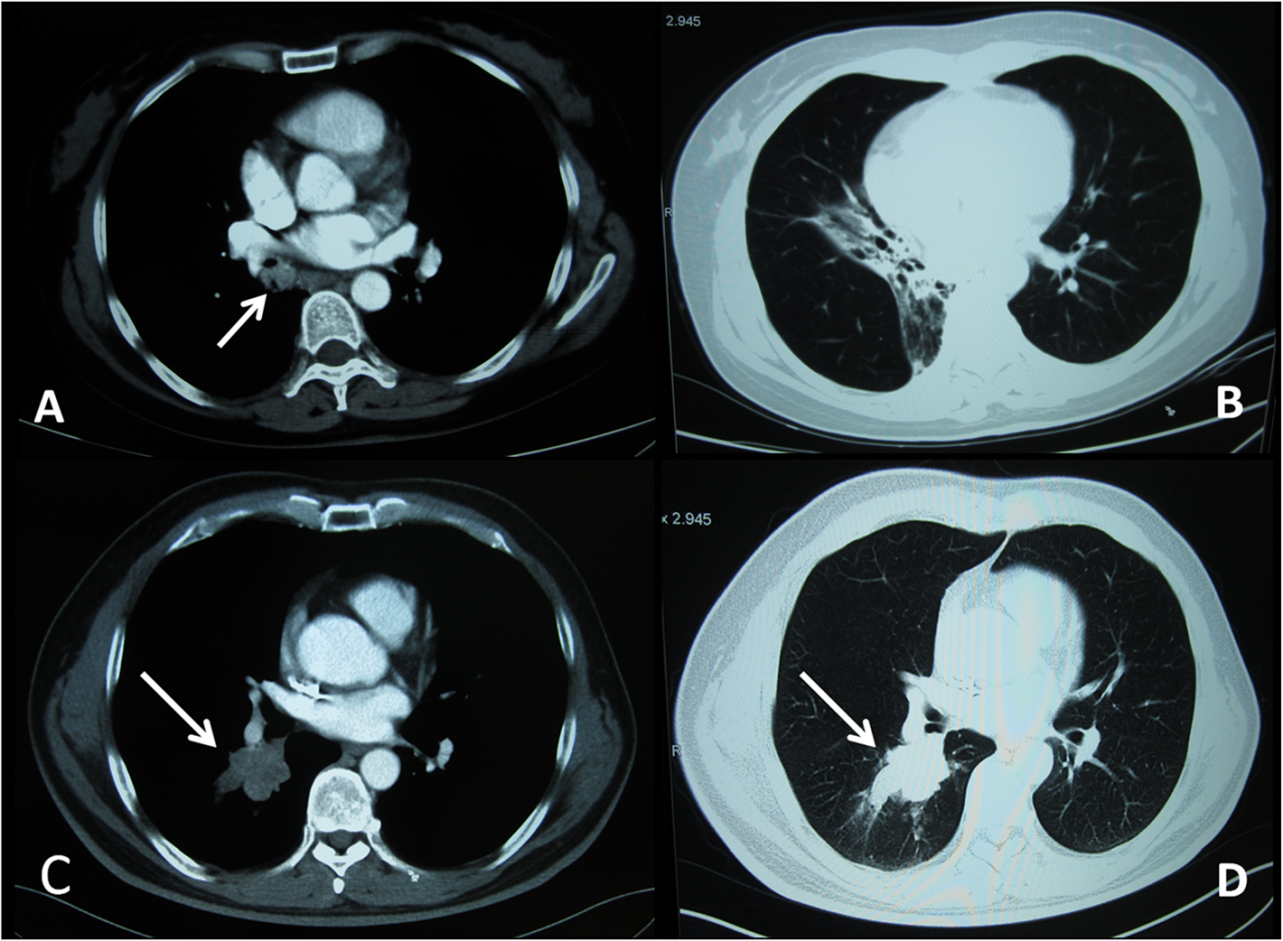
**Computed tomography (CT) features. (A)** CT showed a mass in the middle segmental bronchus of the right lobe (arrowhead). **(B)** Patient with partial atelectasis of the right lower lobe was observed. **(C and D)** CT revealed a mass shadow in the hilus of the right lung of the same patient (arrowhead). CT, computed tomography.

There were four male and four female patients, whose mean age was 50.67 years (35 to 71 years). All four male patients were heavy smokers (mean 43 packs/year (median 45, range 20 to 60)). The most common presenting symptoms were hemoptysis and cough (Table 1). Two tumors were located in the upper lobes and three masses were located in the lower lobes. In three cases, the tumor could be seen as hilar mass and there was only one patient with partial atelectasis (Figure 1A,B,C,D). In the other patients, peripheral nodule (n = 2) and infiltrate (n = 1) were also observed on radiography. The sixth was located in the trachea and the eighth was located in the middle segmental bronchus of the right lobe, which were observed by the fiberbronchoscopy. Three patients who had a preoperative diagnosis of MEC had an endobronchial component and in the other patients (n = 3) the neoplasms were not visible on bronchoscopy. Two patients refused to have the endoscopy for the pain caused by the examination.

**Table 1:** Characteristics of the patients

**Patient**	**Age (years)**	**Gender**	**Packs of cigarettes smoked (per year)**	**Symptoms**	**Fiberbronchoscopy**	**Localization**	**Diameter (cm) CT/surgical**	**Preoperative diagnosis**
1	49	F	-	Hemoptysis	Yes	Right upper lobe	2.5/3.5	NSCLC
2	52	F	-	Cough, dyspnea	Yes	Left main bronchus	1/1.5	MEC
3	71	M	60	Hemoptysis, chest pain	No	Left lower lobe	4.0/5.5	NSCLC
4	69	F	-	Hemoptysis	No	Right lower lobe	3.6/4	NSCLC
5	35	M	20	Hemoptysis, chest pain	Yes	Left upper lobe	1.5/2	NSCLC
6	37	F	-	Cough	Yes	Trachea	1/1.5	MEC
7	47	M	50	-	Yes	Right lower lobe	3.2/4	NSCLC
8	48	M	40	Cough, dyspnea	Yes	Middle segmental bronchus of right lobe	1.2/1.5	MEC

CT, computed tomography; MEC, mucoepidermoid carcinoma; NSCLC, non-small-cell lung cancer.

Altogether, the preoperative microscopical diagnosis was exact in three cases, but in the others (n = 5) NSCLC was diagnosed. No mortality occurred in the early postoperative period. The operations were performed with lobectomy by standard posterolateral thoracotomy in two patients or by VATS in four patients; one with main bronchial sleeve resection and the other one with trachea sleeve resection (Table 2).

**Table 2:** Diagnostic characteristics of the lesions

**Patient**	**Preoperative TNM**	**Preoperative stage**	**Postoperative TNM**	**Postoperative stage**	**Cellular components of end diagnosis**	**Resection**	**Follow-up (months)**	**Prognosis**
1	T2aN2M0	IIIA	T2aN2M0	IIIA	MEC (low grade)	Right upper lobectomy	84	Alive
2	T3N1M0	IIIA	T3N0M0	IIB	MEC (low grade)	Left main bronchial sleeve resection	88	Alive
3	T2aN1M0	IIA	T3N1M0	IIIA	MEC (high grade)	Left lower lobectomy	15	Death
4	T2aN0M0	IB	T2aN0M0	IB	MEC (low grade)	Right lower lobectomy by VATS	44	Alive
5	T1aN0M0	IA	T1aN0M0	IA	MEC (low grade)	Left upper lobectomy by VATS	48	Alive
6	T4N1M0	IIIA	T4N0M0	IIIA	MEC (low grade)	Trachea sleeve resection	36	Alive
7	T2aN0M0	IB	T2aN0M0	IB	MEC (low grade)	Right lower lobectomy by VATS	24	Alive
8	T3N0M0	IIB	T3N1M0	IIIA	MEC (high grade)	Right middle and lower lobectomy by VATS	8	Alive

MEC, mucoepidermoid carcinoma; TNM, TNM classification of malignant tumors; VATS, video-assisted thoracoscopic surgery.

**Figure 2:**
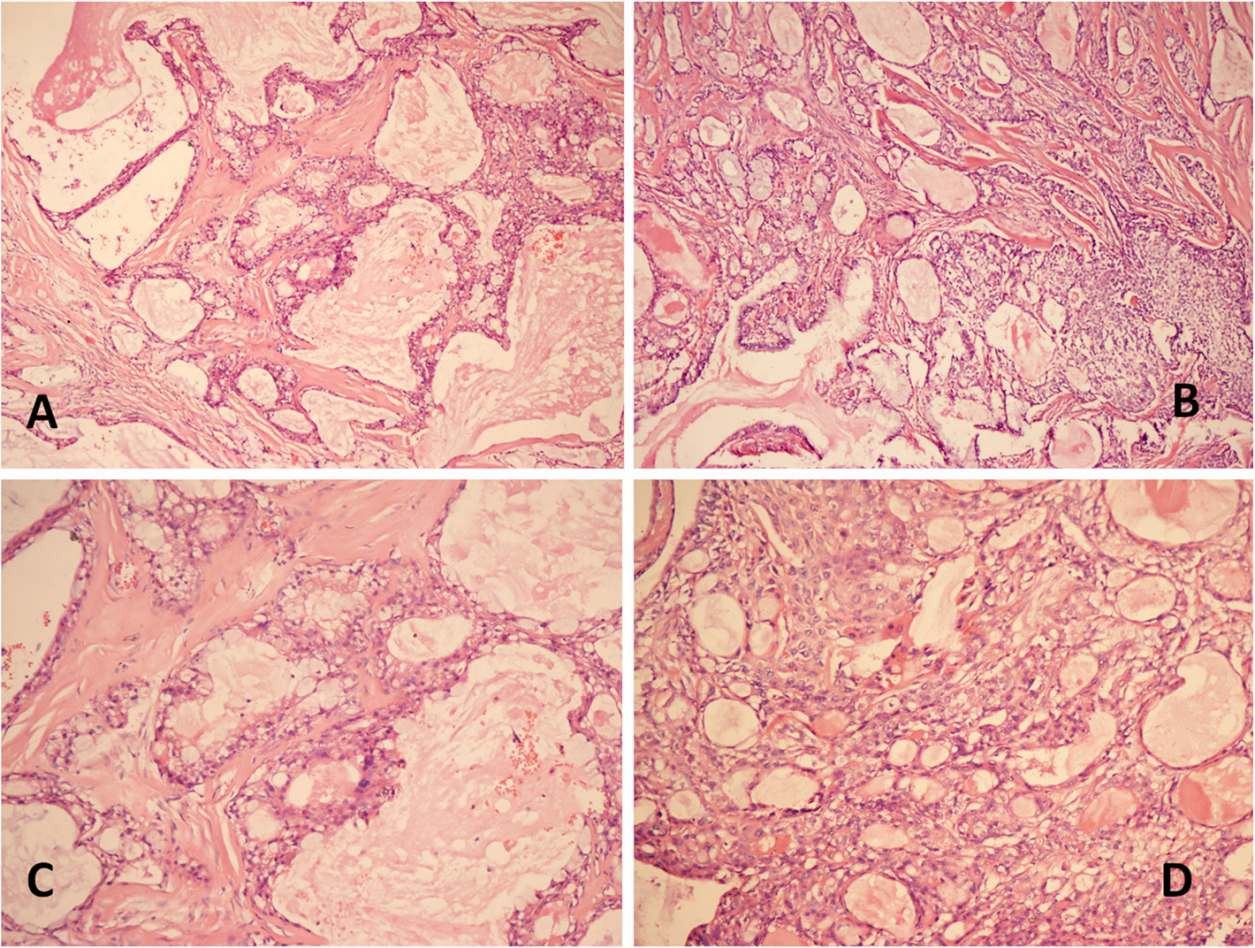
**Histological features. (A and B)** HE staining revealed MEC of the pulmonary neoplasms. (HE × 200). **(C and D)** HE staining revealed MEC of the pulmonary neoplasms. (HE × 400). HE, hematoxylin and eosin; MEC, mucoepidermoid carcinoma.

Pathological confirmation was based on routine light microscopic sections stained by hematoxylin and eosin (HE) (Figure 2). Histology was controlled carefully retrospectively in every case. The characteristics of the tumors were consistent with low grade MEC (n = 6) and high grade MEC (n = 2). All of the patients were sent for oncological evaluations, and three patients with N1 or N2 disease received chemotherapy. One of the patients died from brain metastasis at 15 months. Seven of the eight patients were alive at the time of evaluation. The median survival time was 40 (range 8 to 88) months.

## Discussion

MEC is the most common salivary gland malignancy [6]. Approximately half of tumors occur in the minor salivary gland, particularly in the palate. As a malignant tumor of bronchial gland origin, which was first described by Smetana in 1952 [7], it has a presumed incidence of 0.1% to 0.2% of all lung cancers [8]. They can occur over a broad age range. Recently, however, most cases have been generated from the pediatric population. The mean age in our series, 50.67 years, is considerably older than in most series. Although some studies have reported a male predominance, most reports, including our own, fail to demonstrate a clear predilection based on gender. Smoking is not a major risk factor. In our group, four patients were males and the others were females. There was no difference between them on prevalence with smoking or not. Symptoms are primarily those of bronchial irritation and obstruction, and include cough, wheezing, hemoptysis, and postobstructive pneumonia. Sometimes MEC can mimic that of bronchial asthma [9]. The more worrisome constitutional symptoms of pain, weight loss, and malaise, which were common in the series of Turnbull and colleagues [10], reflect the aggressive potential of these tumors. In our series, there were four patients that showed hemoptysis and the others had cough, chest pain, and dyspnea, as expected.

The majority of MECs arise from bronchial glands in the lumen of a main, lobar, or segmental bronchus, and CT findings are similar to those of bronchial carcinoid tumors. According to Zwiebel *et al*. [11], bronchial carcinoid is also associated with frequent postobstructive pneumonia or atelectasis, and the shape of the tumor is frequently nonspherical, with its long axis parallel to that of the nearest major bronchovascular bundle. It was difficult to predict the exact endobronchial location of the tumors on chest radiographs, especially when they were present at the level of the segmental bronchus. When the tumor manifested as a solitary pulmonary nodule without any other associated findings, the radiographic finding was nonspecific and was not different from that of other benign and malignant nodules. From the CT, however, there were several clues that helped us to determine the endobronchial location of the tumors. As Elnayal *et al*. reported [12], higher FDG uptake is associated with nodal disease in patients with primary salivary gland-type tumors of the lung but is not predictive of survival, whereas CT features suggestive of advanced disease correlate with worse outcome. With all tumors being nonspherical, the direction of the longest diameter of the tumor was parallel to the direction of the branching pattern in the corresponding airways containing the tumor. Associated findings of distal bronchial dilatation with mucoid impaction, postobstructive pneumonia, subsegmental atelectasis, or air trapping were suggestive of an endobronchial mass. Additional ancillary findings that indicated the endobronchial location of the tumors were areas of peripheral lucency or a crescent of air around the tumor. The findings were suggestive of a residual lumen of an ectatic bronchus that was occupied by the endobronchial tumor. In our cases, two tumors were located in the upper lobes and three masses were located in the lower lobes. Two neoplasms were observed in the left main bronchus or middle segmental bronchus of the right lobe, respectively, and one mass was found in the trachea by fiberbronchoscopy. One patient showed partial atelectasis of the right lower lobe.

Foci of calcification or ossification have been reported to be present within the tumor [13]. The incidence of calcification in MEC of the lung was much higher than that of the more common forms of the pulmonary carcinomas. In our series, punctuate calcification within the tumor was not seen in the patients. According to the report by Kim *et al*. [14], the tumor is known to be vascular, and marked enhancement of the tumor has been noted at CT; whereas MEC showed only mild and nonuniform enhancement in our cases.

MEC originates from glandular tissue identical with salivary glands located in the submucosa of the trachea and bronchus. It is included among carcinomas of salivary gland-types along with adenoid cystic carcinoma according to the WHO histological classification of lung cancer [15]. It is characterized by a mixture of mucus-producing, glandular and squamous epithelial cells, as well as intermediate cells with both properties at various percentages, and by various growth patterns such as cystic, papillary, and solid structures. Mucus-producing cells form lumens in some cases. They are classified as high grade or low grade mucoepidermoids on the basis of their histologic appearance. Low grade malignant tumors have mostly cystic components. Microscopic invasion into pulmonary parenchyma is unusual. Mild cytologic atypia can be seen. Metastasis to regional lymph nodes is distinctly unusual. High grade tumors more commonly show areas of solid growth. Atypia, mitotic activity, and necrosis are characteristic and regional lymph node involvement is more frequent in these tumors. This high grade variant can occasionally be difficult to distinguish from adenosquamous carcinoma [16]. Klacsmann *et al*. [17] proposed several criteria by which this differentiation can be made. In mucoepidermoid tumors the surface epithelium does not show carcinoma in situ, and although squamous metaplasia may be present, one would not expect to find atypical metaplasia, except on a coincidental basis. The cells of both solid and glandular areas of the tumor often have a more uniform, almost bland appearance, which differs from that usually seen in bronchogenic carcinoma in which the malignant nuclear features and cytoplasmic variability are usually more pronounced. Large areas of frank keratinization, including abundant individual cell keratinization, is a feature of bronchogenic carcinoma that is not seen in the mucoepidermoid tumor of the lung. In our series, the characteristics of the tumors were consistent with low grade MEC (n = 6) and high grade MEC (n = 2). Tumor cells had a scattering of mucus-producing epithelial components in papillary growth of stratified squamous epithelia with anisokaryosis and minimal pleomorphism, indicating a diagnosis of MEC.

Radical surgery based on lung cancer treatment is performed for MEC, and in recent years this operation has been frequently performed using VATS [18]. MECs of the lung are often treated by lobectomy, sleeve resection, local resection, segmental resection, or even endoscopic removal. In our cases, two patients had the operations of lobectomy by a standard posterolateral thoracotomy and tumors in the other four patients were completely resected by VATS; one with left main bronchial sleeve resection and the other one with trachea sleeve resection.

Most patients with this disease have a favorable outcome after a complete resection [19]. However, the disease may recur in distant organs in a small subset of the patients during long-term follow-up periods. A review of 173 salivary gland MECs [19] noted that distant metastases affected 16 patients (9.2%), most frequently in the lungs. The analogous consequence was reported in some studies [20], which indicated the prognosis of older patients is worse and that hilar lymph node metastasis indicates worse prognosis. In our experience, patients with low grade mucoepidermoids can be expected to be cured following complete resection. On the other hand, however, cases of high grade malignant neoplasm surgery results in significantly worse prognosis. The older patient in our series only survived for 15 months and died from the brain metastasis. It seems that survival correlates with the presence of lymph node metastasis. Nevertheless, the metastasis of low grade MEC of the lung is rare and low grade MECs have a much better prognosis than high grade carcinomas [21]. Patients with low grade MECs generally have a good prognosis, with a 5-year survival rate of 95%, and adjuvant treatment is considered unnecessary. However, effective treatment measures for high grade tumors have not been established, and these cases reportedly have a poor prognosis [22]. Under such circumstances, there are several reports on the efficacy of the tyrosine kinase inhibitor gefitinib in patients with epidermal growth factor receptor (EGFR) gene mutations [23,24], and this molecularly targeted therapy is likely to improve the prognosis of cases with progressive high grade and recurrent MEC.

## Conclusions

MEC of the lung is a rare malignant neoplasm. A preoperative diagnosis of MEC of the lung is difficult for the reason that the tumor can present as a solitary pulmonary peripheral nodule or as a hilar mass from the CT. Complete surgical resection is still the effective treatment for pulmonary MEC. Patients with low grade mucoepidermoids can be expected to be cured following complete resection. However, in cases of high grade malignant neoplasm, surgery results in a significantly worse prognosis. Careful histological typing plays a key role in prediction of late results, and more studies and larger, multicentric series are needed.

### Consent

Written informed consent was obtained from the patients for publication of this case report and any accompanying images. A copy of the written consent is available for review by the Editor-in-Chief of this journal.

## Abbreviations

CT: Computed tomography; EGFR: Epidermal growth factor receptor; HE: Hematoxylin and eosin; MEC: Mucoepidermoid carcinoma; MRI: Magnetic resonance imaging; NSCLC: Non-small-cell lung cancer; TNM: TNM classification of malignant tumors; VATS: Video-assisted thoracoscopic surgery.

## Competing interests

The authors declare that they have no competing interests.

## Authors’ contributions

CS was involved in drafting the manuscript, acquisition of data, and preparation of the figures. GC designed and revised the manuscript. All authors read and approved the final manuscript.
